# Exclusive Enteral Nutrition Exerts Anti-Inflammatory Effects through Modulating Microbiota, Bile Acid Metabolism, and Immune Activities

**DOI:** 10.3390/nu14214463

**Published:** 2022-10-24

**Authors:** Fangfei Xiao, Xuefeng Gao, Hui Hu, Jun Le, Yongheng Chen, Xingsheng Shu, Ziwei Liang, Yang Xu, Yizhong Wang, Ting Zhang

**Affiliations:** 1 Department of Gastroenterology, Hepatology and Nutrition, Shanghai Children’s Hospital, School of Medicine, Shanghai Jiao Tong University, Shanghai 200062, China; 2 Institute of Pediatric Infection, Immunity and Critical Care Medicine, Shanghai Children’s Hospital, School of Medicine, Shanghai Jiao Tong University, Shanghai 200062, China; 3 Central Laboratory, Shenzhen Key Laboratory of Precision Medicine for Hematological Malignancies, International Cancer Center, Shenzhen University General Hospital, Shenzhen 518000, China; 4 Department of Gastroenterology and Hepatology, Shenzhen University General Hospital, Shenzhen 518000, China; 5 Department of Physiology, School of Basic Medical Sciences, Shenzhen University Health Science Center, Shenzhen 518000, China; 6 School of Medicine, Southern University of Science and Technology, Shenzhen 518100, China

**Keywords:** Crohn’s disease, EEN, bile acid, hyocholic acid, gut microbiota

## Abstract

Exclusive enteral nutrition (EEN) can induce remission in patients with pediatric Crohn’s disease (CD). This study aims to depict EEN’s modification of bile acid (BA) metabolism in pediatric CD and explores the effect of the EEN-enriched BA in inhibiting the inflammatory response. The twelve enrolled pediatric CD patients showed BA dysmetabolism, represented by decreased levels of fecal secondary and unconjugated BAs as determined by UPLC–TQMS, which were accompanied by gut microbiota dysbiosis and reduced BA-metabolizing bacteria including *Eubacterium* and *Ruminococcus* genera, assessed by shotgun metagenomic sequencing. EEN treatment induced remission in these patients at eight weeks, and nine patients remained in stable remission for longer than 48 weeks. EEN improved BA dysmetabolism, with some enriched BAs, including hyocholic acid (HCA), α-muricholic acid (αMCA), strongly associated with decreased severity of CD symptoms. These BAs were significantly correlated with the increased abundance of certain bacteria, including *Clostridium innocuum* and *Hungatella hathewayi,* which express 3β-hydroxysteroid dehydrogenase and 5β-reductase. HCA could suppress TNF-α production by CD4+ T cells in the peripheral blood mononuclear cells (PBMCs) of CD patients. Moreover, intraperitoneal injection of HCA could attenuate dextran sulfate sodium (DSS)-induced mouse colitis. Our data suggests that BA modification may contribute to the EEN-induced remission of pediatric CD.

## 1. Introduction

A steadily increasing incidence of inflammatory bowel disease (IBD) worldwide over the past four decades indicates its emergence as a global disease [1,2]. Crohn’s disease (CD) is characterized by transmural inflammation, causing thickening and narrowing of the gastrointestinal tract (GI) wall and eventually leading to the disabling development of deep ulcerations, fistulae, strictures, and abscesses. A defect in barrier integrity is closely associated with profound alterations in the intestinal metabolome, including a shortage of short-chain fatty acids (SCFA) [3], altered concentrations of amino acids and lipid acids [4], and dysregulation of bile acid (BA) pool composition [5]. These metabolic abnormalities are proved to be partially driven by gut microbiota dysbiosis [6,7,8,9].

Clinical studies [10,11] and meta-analyses [12,13] demonstrated that exclusive enteral nutrition (EEN) was equivalent to corticosteroids for induction of remission and mucosal healing in children and adolescents with active CD, and had fewer side effects. In addition, EEN has the advantages of improving nutritional indices, managing IBD-related complications such as abscesses, fistulae and stenosis, and facilitating avoidance of surgery [14]. Accordingly, consensus guidelines of the European Crohn’s and Colitis Organization (ECCO)/European Society for Pediatric Gastroenterology, Hepatology and Nutrition (ESPGHAN) recommend using EEN for 6–8 weeks as the initial induction of remission therapy in mild to moderate active pediatric CD [15]. The mechanisms by which EEN attenuates physiological markers of inflammation and promotes mucosal healing in CD are yet to be elucidated. Nevertheless, growing evidence supports that EEN works by modifying gut microbial communities and host–microbiota co-metabolism within the gut [16,17].

BAs, especially secondary BAs, play an important role in modulating the inflammatory responses within the gut. In CD, a deficit of active BA transporters in the ileum is responsible for BA malabsorption and consequently results in increased fecal loss and an altered BA pool composition, such as higher conjugated and lower secondary BA concentrations [5]. Deconjugated BAs and secondary BAs can interact with nuclear receptors, including the farnesoid X receptor (FXR), G protein-coupled bile acid receptor 1 (TGR5), vitamin D receptor (VDR), and retinoic acid-related orphan receptor-γt (RORγt), which have been shown to have protective functions in CD by inhibiting intestinal inflammation and preserving epithelial barrier integrity [18,19,20]. Two recent animal studies showed that BAs could regulate the ratio of anti-inflammatory regulatory T cells (Tregs) to proinflammatory effector helper T cells (specifically Th17) [21,22]. In specific, 3-oxo-LCA (3-keto-LCA) can bind RORγt to inhibit the differentiation of Th17 cells, thereby attenuating intestinal inflammation. These data suggest an important immune regulatory role of microbial BA metabolites in controlling inflammation.

In this context, we hypothesized that EEN modifies BA pool composition, thereby regulating intestinal immune responses and enhancing remission in pediatric CD patients. We conducted a longitudinal observational study of EEN therapy in twelve pediatric CD patients. Fecal samples were collected prior to and after EEN for analysis of fecal microbiota with shotgun metagenomic sequencing and quantification of fecal BA using ultra-performance liquid chromatography coupled with triple quadrupole mass spectrometry (UPLC–TQMS). All patients achieved remission after eight weeks of EEN induction therapy. We identified multiple BAs associated with the therapeutic outcomes of EEN, and their lives were significantly correlated with the relative abundance of several gut bacteria that express important BA-metabolizing enzymes. Among the EEN-enriched BAs, we explored and demonstrated the anti-inflammatory properties of hyocholic acid (HCA) both in vitro with peripheral blood mononuclear cells (PBMCs) isolated from CD patients, and in vivo with a dextran sulfate sodium (DSS)-induced mouse colitis model.

## 2. Materials and Methods

### 2.1. Study Design, Subjects, and Sample Collection

The study was approved by the Institutional Review Board of Shanghai Children’s Hospital (approval No. 2020R034-E02) and adhered to the tenets of the Declaration of Helsinki. Twelve pediatric CD patients, aged from 6–17 years, participated in this longitudinal observational study for EEN therapy during June 2016–May 2020 at Shanghai Children’s Hospital. The diagnosis of CD was confirmed based on established clinical criteria and a combination of endoscopic, histological, and radiological criteria and/or biochemical investigations [23]. All patients received EEN administrated via oral route or nasogastric tube.

Patients’ demographics, disease location, laboratory parameters, and medications were recorded at baseline (BSL), four weeks, and eight weeks of EEN. Sixteen healthy subjects age- and gender-matched to the CD patients, without a previous history of chronic disease, allergies, or medications (antibiotics in particular) within the past six months, were enrolled in the study as healthy controls (HC). Each HC provided a single stool. A total of 29 fecal samples were collected from 12 pediatric CD patients, including 10, 8, and 11 samples at BSL, four weeks, and eight weeks of EEN treatment, respectively. All stool samples were stored at −80 °C immediately after collection.

### 2.2. DNA Preparation, Metagenomic Sequencing, and Taxonomic Annotation

Genomic DNA was extracted using the QIAamp DNA Stool Mini Kit (Qiagen, Hilden, Germany) combined with the bead-beating method, as per the manufacturer’s recommendations. DNA quantity was determined using the BR dsDNA Qubit Assay kit (Invitrogen, Carlsbad, CA, USA) according to the manufacturer’s instructions. Sequencing libraries were constructed using the NEBNext Ultra DNA Library Prep Kit for Illumina (NEB, Ipswich, MA, USA). Briefly, 1 µg of genomic DNA was sheared to obtain an average fragment size of 350 bp using a sonicator (Covaris, Woburn, MA, USA). The fragments were end-polished, A-tailed, and ligated with a full-length adapter for Illumina sequencing, followed by further polymerase chain reaction (PCR) amplification. Libraries were pooled and diluted to 2 ng/μL, analyzed for molecular length using an Agilent 2100 Bioanalyzer, and quantified using real-time PCR. Following library preparation, sequencing was performed on an Illumina NovaSeq 6000 platform (Illumina, USA) via the PE150 strategy.

The adaptor was trimmed using Trim Galore v.0.6.5 (https://github.com/FelixKrueger/TrimGalore, accessed on 19 November 2019), after which Fastp [24] was used to filter reads with more than 10 N bases, Q20 < 20%, or length shorter than 60 bp. Bowtie2 [25] was used to further filter human genomic reads by mapping the metagenomic reads against the latest human genome reference (hg19) [26]. MetaPhlAn3 [27] was applied to profile the composition of microbial communities determined from metagenomic shotgun sequencing, and taxonomic annotation was generated using version v20 of the database with default parameters.

### 2.3. Analysis of Fecal Microbiota with Shotgun Metagenomic Sequencing

MicrobiomeAnalyst [28] was used to analyze the bacterial community composition data [29]. Data with low count (minimum count < 4 and prevalence in samples < 20%) and low variance (less than 10% inter-quantile range measure of variance) were filtered and were further normalized based on centered log-ratio (*clr*) transformation to account for the compositional nature of the metagenomic data. Microbiome alpha diversity was measured using the Shannon’s, Chao1, Simpson’s, and Inverse Simpson’s indexes. The beta diversity among samples was calculated through principal coordinate analysis (PCoA) to Euclidean distance based on *clr*. Permutational multivariate analysis of variance (PERMANOVA) was carried out to test whether the gut microbiome structure was significantly different. Statistical differences in alpha diversity and abundances of taxa were assessed via Mann–Whitney U tests and Kruskal–Wallis tests, with *p*-values adjusted using the Benjamini–Hochberg correction procedure for controlling the false discovery rate (FDR).

### 2.4. Quantification of Fecal Bile Acids with Ultra-Performance Liquid Chromatography Coupled with Triple Quadrupole Mass Spectrometry

Fecal samples were kept frozen at −80 °C and then lyophilized overnight in a freeze dryer system (Labconco FreeZone 2.5 Plus, Kansas City, MO, USA). BAs were extracted using a two-step extraction procedure as previously described [30,31]. In brief, an aliquot of 10 mg of lyophilized feces from each sample was homogenized in 20 mL of deionized water. Then, 180 μL of acetonitrile:methanol = 80:20 containing the six internal standards (CA-d4, GCA-d4, GCDCA-d4, GDCA-d4, LCA-d4, and UDCA-d4 at 50 nM each) was added, homogenized for 5 min, and centrifuged at 13,200 rpm at 4 °C for 15 min. After transferring the supernatant to a 1.5 mL Eppendorf tube, the residue was further extracted by adding 180 μL acetonitrile:methanol = 80:20 containing the six internal standards as the first extraction solvent. The two supernatants were combined and vortexed for 3 min before centrifugation at 13,200 rpm at 4 °C for 15 min.

BA analysis was performed via the UPLC–TQMS system. The elution solvents were water + 0.01% formic acid (A) and acetonitrile:methanol = 19:1 + 0.01% formic acid (B). A total of 46 types of BAs were separated on a reverse column by gradient elution over 20 min at a flow rate of 450 μL/min as follows: 0–2 min (20% B), 2–3 min (20–25% B), 3–6 min (25% B), 6–8 min (25–35% B), 8–11.5 min (35% B), 11.5–18 min (35–99% B), 18–19 min (99% B), and 19–20 min (99–20% B). Mass spectrometry (MS) was performed in negative electrospray ionization mode. The cone voltage and collision energy were optimized for each BA using a QuanOptimize application manager (Waters Corp., Milford, MA, USA).

MetaboAnalyst 5.0 [32] was used for statistics and feature selection of the metabolomic data. Univariate statistical analysis was performed using Mann–Whitney U tests and Kruskal–Wallis tests with *p*-values adjusted using the Benjamini–Hochberg correction procedure for controlling FDR.

### 2.5. Peripheral Blood Mononuclear Cell Isolation and Stimulation

Heparin whole blood was collected from five adult treatment-naïve CD patients. PBMCs were isolated from the whole blood by centrifugation using Biocoll Separating Solution (BiochromGmbH, Berlin, Germany). All samples were incubated for 4 h in a medium consisting of RPMI with 10% fetal bovine serum and 1% penicillin–streptomycin (all three: Gibco, Carlsbad, CA, USA) at 37 °C in HERA Cell 150 (Thermo Fisher Scientific, Waltham, MA, USA) after adding CD3/CD28-coated magnetic beads (1 μg/mL). In addition, Brefeldin A (Sigma-Aldrich, St. Louis, MO, USA) dissolved in dimethyl sulfoxide (Sigma-Aldrich) was added to all flow cytometry samples after 2 h to prevent cells from secreting tumor necrosis factor-α (TNF-α) and interferon-γ (IFN-γ).

### 2.6. Flow Cytometry

For flow cytometry analysis, single-cell suspensions were stained with the following antibodies: BV605 anti-mouse CD4 and Alexa Fluor 700 CD8 (Biolegend, San Diego, CA, USA). For intracellular staining, cells were stimulated for 4 h at room temperature with PMA (50 ng/mL), ionomycin (1 µg/mL), and Brefeldin A. Following fixation and permeabilization, cells were stained with Alexa Fluor 647 anti-human IFN-γ and PE anti-human TNF-α (Biolegend, San Diego, CA, USA). Cells were acquired with a NovoCyte Quanteon (Agilent Technologies, Santa Clara, CA, USA), and data analysis was performed with FlowJo software (Version 10.8, FlowJo, Ashland, OR, USA).

### 2.7. Enzyme-Linked Immunosorbent Assay

The cytokine production from PBMCs was assessed using ELISA assay (BioGems, Westlake Village, CA, USA) following the manufacturer’s instructions. Supernatant and standard controls (100 μL prepared according to kit instructions) were transferred to a pre-coated primary antibody (IFN-γ or TNF-α) stripwell microplate, incubated at 37 °C for 90 min, and washed. The avidin–biotin–peroxidase complex was added at 100 µL/well and incubated at 37 °C for 30 min. After additional washes, 90 µL of color developing reagent was added to each well and incubated in the dark for 30 min. The reaction was terminated by the addition of 100 µL stop solution, and O.D. absorbance was read with a microplate reader at 450 nm.

### 2.8. Dextran Sulfate Sodium Colitis Model

C57/BL6 male mice (6–7 weeks old) were purchased from GemPharmatech (Nanjing, China). For acute colitis induction, mice were administered 3% (*w*/*v*) DSS (36–50 kDa; MP Biomedicals, Santa Ana, CA, USA) in drinking water for nine days. For the treatment groups, HCA (30 mg/kg body weight, Sigma-Aldrich, St. Louis, MO, USA) was intraperitoneally injected from day four to day nine. Saline was injected as the vehicle in the control group. Body weight was monitored daily to assess the severity of intestinal colitis. The colon length was measured at sacrifice, which was performed on day nine. The animal experimental procedures were approved by the Laboratory Animal Ethics Committee of Shenzhen University.

### 2.9. Statistical Analysis

Continuous variables were presented as medians with interquartile range (IQRs, 25th to 75th percentiles), and categorical variables were summarized as absolute numbers with percentages. SPSS software (version 20.0) was used for statistical analyses. Nonparametric Mann–Whitney U tests and Kruskal–Wallis tests were used to analyze continuous variables between two groups and among multiple groups, respectively. The categorical variables were compared using the chi-squared test. The *p*-values were adjusted using the Benjamini-Hochberg correction procedure for controlling FDR. Statistical significance was defined by a *p*-value of less than 0.05.

## 3. Results

### 3.1. Characteristics and Responses of Pediatric CD to EEN Therapy

Demographic and clinical characteristics of the 12 patients that participated in this study are presented in Table 1. Among these patients (eight boys and four girls), the median age at BSL was 13 years (IQR, 11–13 years). Disease location within the GI tract was similar across all patients, with the majority (10/12) having ileocolonic disease (L3). Four patients had comorbidities, including three with perianal fistula and one with hypothyroidism. Ten patients received EEN via oral administration, while two were treated via nasogastric tube. After eight weeks of EEN therapy, significant decreases in erythrocyte sedimentation rate (ESR), white blood cells (WBC), platelet count (PLT), C-reactive protein (CRP), and fecal calprotectin (FCP) were observed (baseline vs. 8 W: 109.5 (93.3, 120) mm/h vs. 23 (9, 23) mm/h, *p* < 0.0001, *q* = 0.0004 for ESR; 11.9 (7.6, 13.4) × 10^9^/L vs. 6.3 (5.0, 7.9) × 10^9^/L, *p* = 0.0014, *q* = 0.0025 for WBC; 522.53 (479.8, 552.8) × 10^9^/L vs. 324 (261.3, 352.5) × 10^9^/L, *p* = 0012, *q* = 0.0025 for PLT; 42 (26,73.5) mg/L vs. 5 (5, 5) mg/L, *p* < 0.0001, *q* = 0.0004 for CRP; 1800 (1800, 1800) μg/g vs. 410 (264.5, 1275) μg/g, *p* = 0.032, *q* = 0.045 for FCP) (Table 1). The hemoglobin (HB), albumin (ALB), and prealbumin (PA) levels significantly increased after EEN therapy (baseline vs. 8 W: 98 (86.8, 105) g/L vs. 117.5 (111.3, 126) g/L, *p* = 0.0013, *q* = 0.003 for HB; 31.5 (24, 34.7) g/L vs. 41.11 (40.5, 44.2) g/L, *p* < 0.0001, *q* = 0.0004 for ALB; 73 (53.1, 130.6) mg/dL vs. 216 (170.6, 256.6) mg/dL, *p* = 0015, *q* = 0.003 for PA) (Table 1). However, there was no significant change in BMI (baseline vs. 8 W: 13.7 (12.8, 15.4) vs. 15.4 (13.8, 18.0), *p* = 0.1135, *q* = 0.1314) and Screening Tool for the Assessment of Malnutrition in Pediatrics (STAMP) score (baseline vs. 8 W: 4.50 (2, 6.50) vs. 3 (3, 6), *p* = 0.1135, *q* = 0.1314) (Table 1).

**Figure 1:**
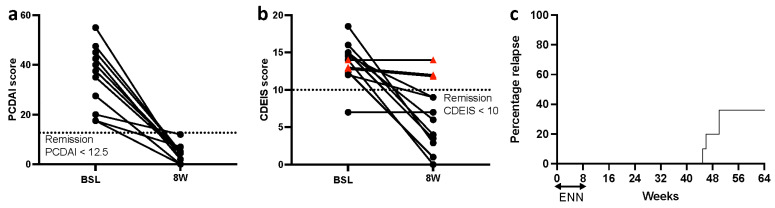
Pediatric CD patients achieved remission after ENN induction therapy. (**a**) PCDAI and (**b**) CDEIS scores at baseline and at week eight of EEN in 12 CD patients. (**c**) Kaplan–Meier survival curve showing time to relapse in 12 CD patients who remained in remission after EEN treatment. CD, Crohn’s disease; EEN, exclusive enteral nutrition; PCDAI, Pediatric Crohn’s Disease Activity Index; CDEIS, Crohn’s Disease Endoscopic Index of Severity.

All patients achieved remission (PCDAI scores < 12) after eight weeks of EEN induction therapy (baseline vs. 8 W: 38.8 (21.9, 44.4) vs. 4.5 (2, 5), *p* < 0.0001, *q* = 0.0004), and the Crohn’s Disease Index of Severity (CDEIS) scores of eight patients dropped below 10 (baseline vs. 8 W: 14 (13, 15) vs. 7 (3.8, 12), *p* < 0.0008, *q* = 0.0025) (Table 1; Figure 1b). Mucosal healing was observed through endoscopy (Supplementary Figure S1). During follow-up assessments, 3 out of 12 (25%) patients subsequently relapsed by week 48 (Figure 1b) and received re-induction treatment of steroids or infliximab. Nine patients remained in remission throughout the study period (>48 weeks).

### 3.2. Gut Microbiota Dysbiosis and Bile Acid Dysmetabolism in Pediatric CD Patients

**Figure 2:**
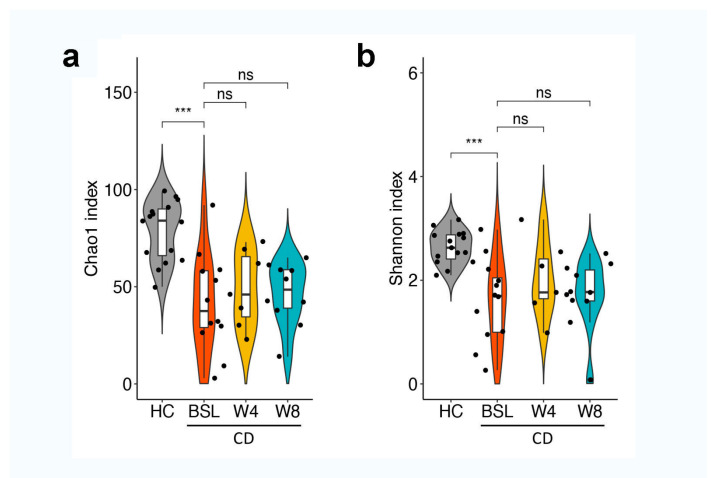
Alpha diversity in healthy children and pediatric CD patients before and after four and eight weeks of EEN treatment. Alpha diversity was measured using the Chao1 index (**a**) and Shannon index (**b**). Statistical differences were assessed via Mann–Whitney U tests and Kruskal–Wallis tests with *p*-values adjusted with the Benjamini–Hochberg correction procedure for controlling false discovery rate. *** *p* < 0.001. HC, healthy controls; CD, Crohn’s disease; BSL, baseline.

**Figure 3:**
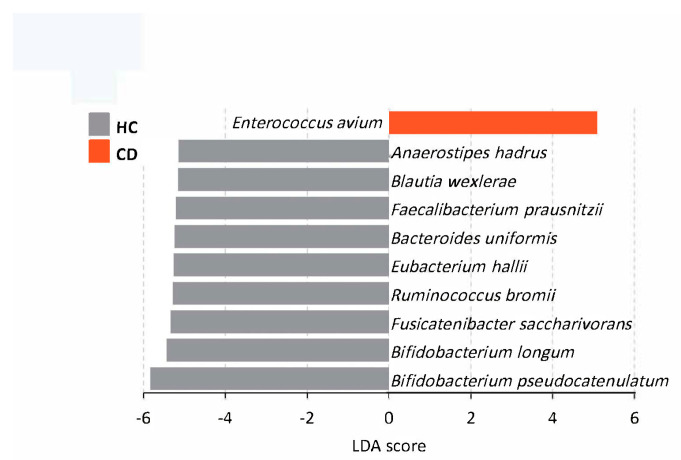
LEfSe identified representative species in healthy controls and CD patients at baseline. Species that fell below FDR < 0.05 and log LDA cutoff ≥ 5.0 were considered significant for feature level comparisons. HC, healthy controls; CD, Crohn’s disease.

The enrolled pediatric patients with CD showed an altered gut microbiota profile compared to healthy individuals, which was characterized by a reduction in biodiversity (Figure 2). Decreased abundance at the bacterial species level, including *Anaerostipes hadrus*, *Blautia wexlerae*, *Faecalibacterium prausnitzii*, *Bacteroides uniformis*, *Eubacterium hallii*, *Ruminococcus bromii*, *Fusicatenibacter saccharivorans*, *Bifidobacterium longum*, and *Bifidobacterium pseudocatenulatum* (Figure 3), and enrichment of genera *Escherichia, Peptostreptococcus,* and *Morganella*, which are known to contain a variety of opportunistic pathogens, were observed in pediatric CD patients (Supplementary Figure S2).

**Figure 4:**
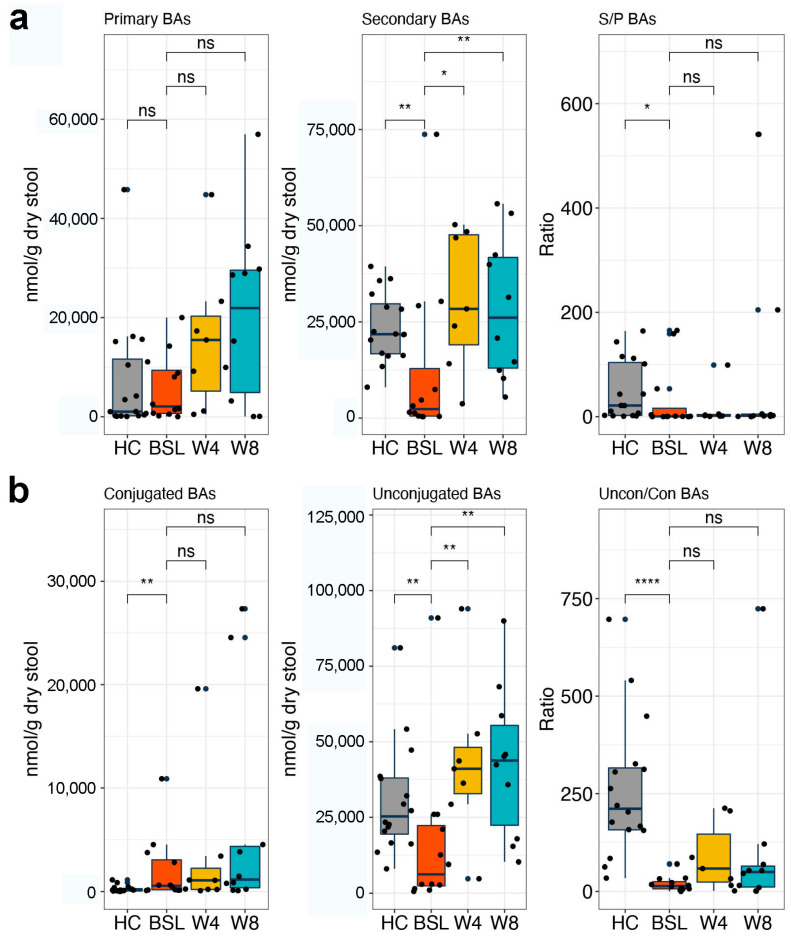
Analysis of fecal bile acid concentrations in pediatric CD patients during EEN induction therapy. (**a**) Primary and secondary BA levels, and ratio of secondary to primary BAs. (**b**) Conjugated and unconjugated BA levels, and ratio of unconjugated to conjugated BAs. Significance was determined via Mann–Whitney U test. * *p* < 0.05; ** *p* < 0.01; **** *p* < 0.0001. BAs, bile acids; HC, healthy controls; BSL, baseline.

**Figure 5:**
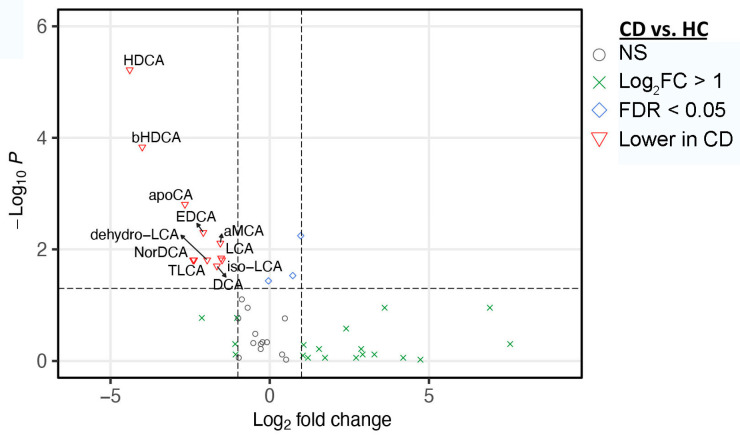
Significant differences in fecal BAs between CD and HC. Volcano plot represents the difference in fecal BAs between CD and HC at baseline. Statistical significance was determined using the two-tailed Mann Whitney U–test with FDR < 0.05 and fold difference > 2.0. HC, healthy controls; CD, Crohn’s disease; FDR, false discovery rate; HDCA, α-hyodeoxycholic acid; bHDCA, β-hyodeoxycholic acid; apoCA, apocholic acid; EDCA, 3-dpideoxycholic acid; aMCA, α-muricholic acid; LCA, lithocholic acid; dehydro-LCA, dehydrolithocholic acid; isoLCA, isolithocholic acid; DCA, deoxycholic acid; NorDCA, 23-nordeoxycholic acid; TLCA, taurolithocholic acid.

By analyzing fecal BA profiles, we found that pediatric CD patients had dysregulated fecal BA profiles that featured reduced levels of secondary and unconjugated BAs and an increased level of conjugated BAs (Figure 4). Moreover, the ratios of secondary/primary BAs and unconjugated/conjugated BAs were also significantly decreased in CD samples (Figure 4). In addition, pediatric CD patients had decreased levels of α-hyodeoxycholic acid (HDCA), β-hyodeoxycholic acid (βHDCA), apocholic acid (apoCA), 3-epideoxycholic acid (EDCA), α-muricholic acid (αMCA), dehydrolithocholic acid (dehydroLCA), 23-nordeoxycholic acid (NorDCA), taurolithocholic acid (TLCA), lithocholic acid (LCA), isolithocholic acid (isoLCA), and deoxycholic acid (DAC) (Figure 5).

### 3.3. EEN Induced CD Remission Is Associated with Coordinated Modifications in Gut Microbiota Composition and Bile Acid Metabolism

**Figure 6:**
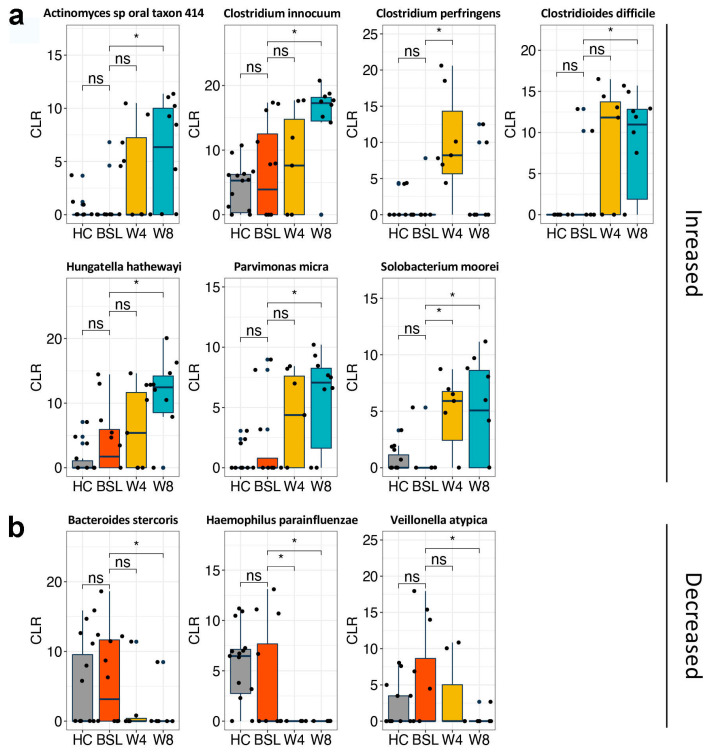
Metagenomic analysis of gut microbiota profiles in CD patients before and after EEN induction therapy. Boxplots demonstrate significantly increased (**a**) and decreased (**b**) bacterial species after either 4 w or 8 w of EEN therapy. Significance was determined via Welch’s *t*-test based on CLR-transformed counts. * *p* < 0.05. HC, healthy controls; BSL, baseline; CLR, centered log-ratio.

Significant changes in gut microbiota composition, but not in *alpha* diversity, were observed after EEN treatment (Figure 2 and Figure 6). EENtreated CD patients showed increased relative abundance of *Hungatella*, *Parvimonas*, *Clostridioides*, *Solobacterium*, *Clostridium*, and *Enterococcus* genera after four or eight weeks of EEN (Supplementary Figure S2). At the species level, increased abundance of *Actinomyces sp oral taxon 414*, *Clostridium innocuum*, *Clostridium perfringens*, *Clostridioides difficile*, *Hungatella hathewayi*, *Parvimonas micra*, and *Solobacterium moorei*, and decreased levels of *Bacteroides stercoris*, *Haemophilus parainfluenzae*, and *Veillonella atypica* were detected after four or eight weeks of EEN treatment (Figure 6). It is worth noting that neither toxin (TcdA and TcdB) was detected in the stool samples, nor were diarrhea or infection symptoms observed in these CD patients.

**Figure 7:**
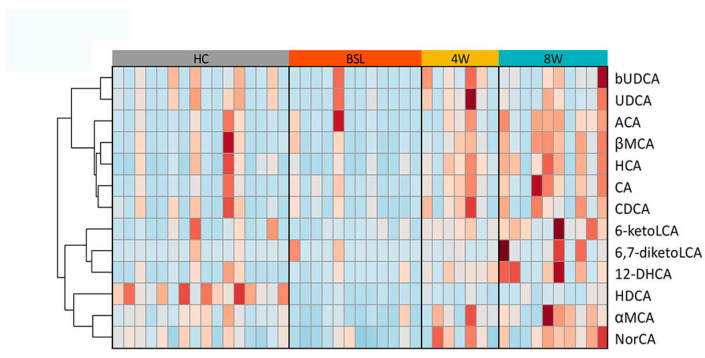
Significantly changed BAs in CD patients after EEN therapy. Heatmap demonstrating significantly changed BAs (all upregulated) in CD patients after EEN induction therapy with fold changes > 2.0. Statistical significance was determined using two-tailed Mann–Whitney U-tests, with FDR < 0.05 and fold difference > 2.0. bUDCA, 3β-ursodeoxycholic acid; UDCA, ursodeoxycholic acid; ACA, allocholic acid; βMCA, β-muricholic acid; HCA, hyocholic acid; CA, cholic acid; CDCA, chenodeoxycholic acid; 6-ketoLCA, 6-ketolithocholic acid; 6,7-diketoLCA, 6,7-diketolithocholic acid; 12-DHCA, 12-dehydrocholic acid; HDCA, α-hyodeoxycholic acid; aMCA, α-muricholic acid; NorCA, norcholic acid.

EEN treatment induced remarkable changes in fecal BA metabolism in pediatric CD patients. Both primary and secondary BA levels increased after EEN treatment, but statistical significance was only achieved for secondary BAs (Figure 4a). However, the ratio of secondary to primary BAs did not significantly change after EEN treatment. The level of unconjugated BAs as well as the ratio of unconjugated to conjugated BAs increased significantly at four weeks post EEN treatment and maintained throughout the eight weeks of the therapeutic period (Figure 4b). Among the significantly increased BAs, the levels of HCA, αMCA, and 6-keto-lithocholic acid (6-ketoLCA) were elevated after four weeks of EEN in the pediatric CD patients, and persisted for eight weeks after EEN therapy (Mann–Whitney U-test, *q* < 0.05 and fold change > 2.0; Figure 7). Most notable is the higher concentration of HCA compared to the other BAs at week eight (mean ± sd, 123.88 ± 47.57 nmol/g). In addition, HDCA, one of HCA species, was also significantly enriched after eight weeks of EEN (Mann–Whitney U-test, *q* < 0.05 and fold change > 2.0; Figure 7).

**Figure 8:**
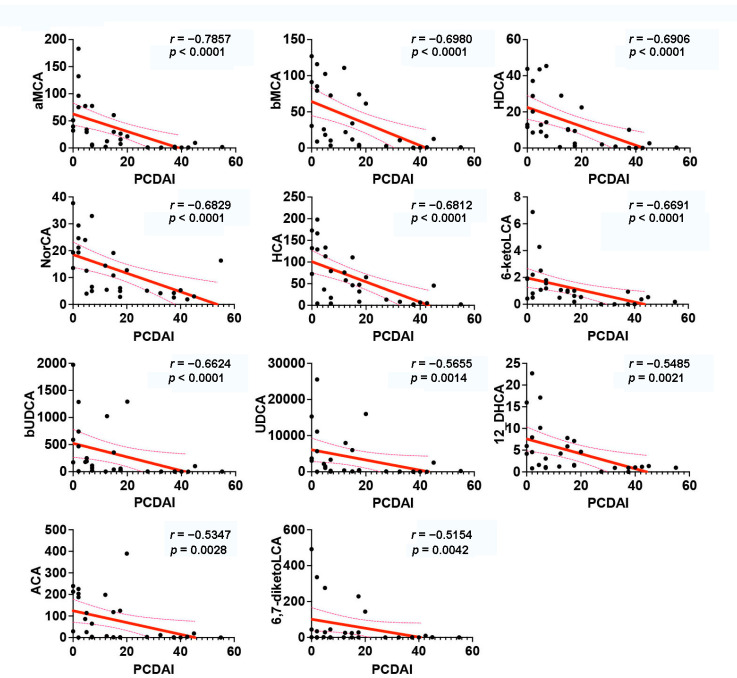
Interrelationships between bile acid profiles and PCDAI in CD. Significant correlations were identified by Spearman correlations with *p* < 0.05 and |*r*| > 0.5. CD, Crohn’s disease; PCDAI, Pediatric Crohn’s Disease Activity Index; aMCA, α-muricholic acid; βMCA, β-muricholic acid; HDCA, α-hyodeoxycholic acid; NorCA, norcholic acid; HCA, hyocholic acid; 6-ketoLCA, 6-ketolithocholic acid; bUDCA, 3β-ursodeoxycholic acid; UDCA, ursodeoxycholic acid; 12-DHCA, 12-dehydrocholic acid; ACA, allocholic acid; 6,7-diketoLCA, 6,7-diketolithocholic acid.

**Figure 9:**
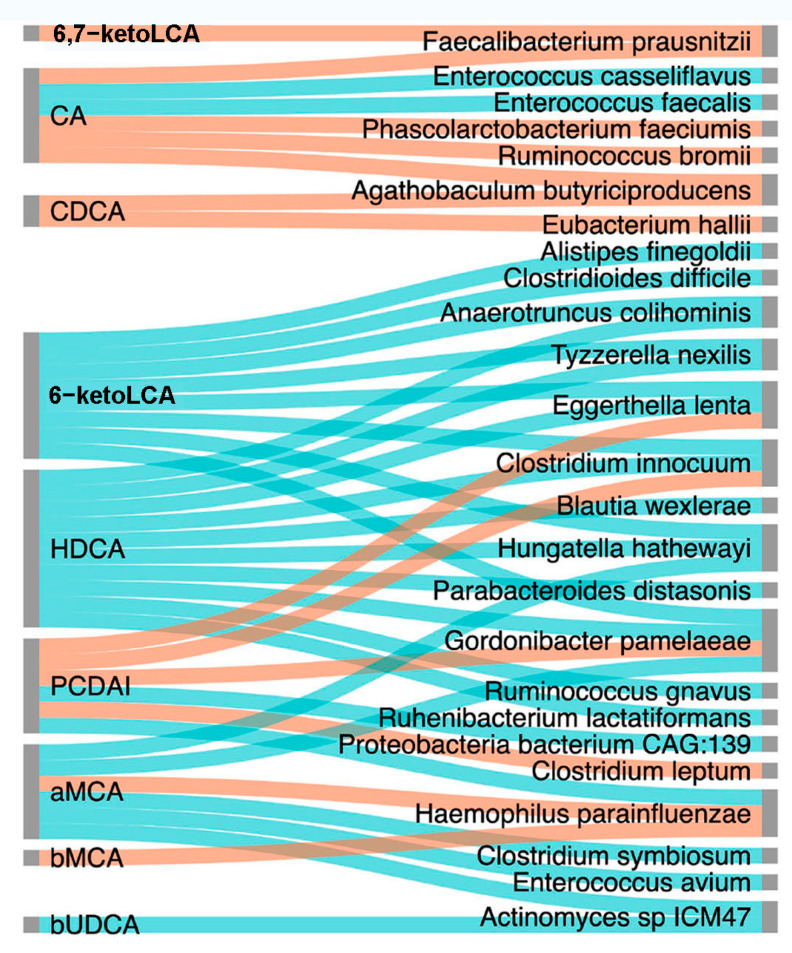
Interrelationships among bile acid profiles, PCDAI and gut microbiota. Sankey plot constructed using NAMAP with Spearman’s rank correlations between PCDAI, gut microbiota (species level), and fecal BAs that were significantly enriched after EEN induction therapy. The network shows associations that are statistically significant with a cutoff of *p* < 0.05 and *r* > 0.7 based on bootstrapping of 100 iterations. Direct correlations are indicated as blue edges and inverse correlations as red edges. 6,7-ketoLCA, 6,7-ketolithocholic acid; CA, cholic acid; CDCA, chenodeoxycholic acid; 6-ketoLCA, 6-ketolithocholic acid; HDCA, α-hyodeoxycholic acid; PCDAI, Pediatric Crohn’s Disease Activity Index; aMCA, α-muricholic acid; βMCA, β-muricholic acid; bUDCA, 3β-ursodeoxycholic acid.

Further analysis indicated that the simultaneous changes in gut microbiota and BA metabolism were closely associated with CD symptom improvement and severity reduction induced by EEN treatment (Figure 8, Figure 9 and Figure S2). The increased levels of HCA, HDCA, αMCA, βMCA, NorCA, 6-ketoLCA, 3β-ursodeoxycholic acid (bUDCA), UDCA, 12-dehydrocholic acid (12-DHCA), allocholic acid (ACA), and 6,7-diketolithocholic acid (6,7-diketoLCA) showed a negative correlation with decreased PCDAI scores (*p* < 0.05 and Spearman correlation coefficient *r* < −0.5; Figure 8). In addition, these BAs were also negatively correlated with FCP, WBC, PLT, CRP, CDEIS, and ESR, indicating their potential contributions in improving CD symptoms (Supplementary Figure S3a). The relative abundance of *Eggerthella lenta*, *Clostridium innocuum*, and *Gordonibacter pamelaeae* inversely correlated with levels of PCDAI, FCP, WBC, and ESR (Supplementary Figure S3b). *Peptostreptococcus stomatis* and *Anaerostipes caccae* were positively correlated with ESR, and *Peptostreptococcus stomatis* was also positively correlated with PCDAI, suggesting a pathogenic/pro-inflammatory potential in these species.

We next tried to identify associations between changed gut bacteria and BAs in CD patients using Spearman’s rank correlation coefficient. The results show that certain gut bacteria were correlated with eight kinds of BAs that increased in CD patients after EEN therapy. For example, *Hungatella hathewayi* showed positive correlations with HDCA, 6-ketoLCA, and αMCA, *Haemophilus parainfluenzae* showed negative correlations with αMCA and βMCA, and *Actinomyces sp ICM47* positively correlated with bUDCA and αMCA. Notably, there was a relative abundance of gut bacteria that were negatively corrected with PCDAI, including *Eggerthella lenta*, *Clostridium innocuum*, and *Gordonibacter pamelaeae*, which were positively correlated with levels of HDCA (Figure 9).

### 3.4. HCA Suppresses TNF Production in CD4 T Lymphocytes within Peripheral Blood of CD Patients

**Figure 10:**
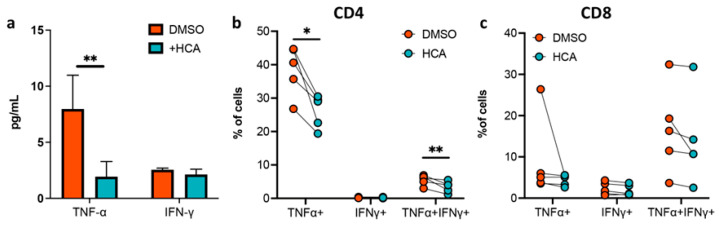
HCA suppresses the inflammatory response in PBMCs isolated from CD patients. (**a**) TNF-α and IFN-γ production was measured via ELISA and compared between HCA- and DMSO-treated PBMCs (isolated from seven CD patients) after stimulation with CD3/CD28-coated magnetic beads. Proportions of TNF-α+ and IFN-γ+ subsets in (**b**) CD4+ and (**c**) CD8+ T cells measured by flow cytometry. The paired samples *t*-test was used to compare the two groups. * *p* < 0.015; ** *p* < 0.01. CD, Crohn’s disease; PBMCs, peripheral blood mononuclear cells; TNF-α, tumor necrosis factor-α; IFN-γ, interferon-γ.

We hypothesized that specific EEN-enriched BAs could contribute to CD remission by suppressing intestinal inflammatory immune responses. To test this hypothesis, we further analyzed the immunity modulation effects of HCA in vitro. PBMCs isolated from five CD patients, and their characteristics and clinical information were included in Supplementary Table S1. The addition of HCA significantly suppressed TNF-α production in PBMCs, while not affecting the levels of IFN-γ (Figure 10a). Moreover, flow cytometry analysis demonstrated that the incidences of IFN-γ- and TNF-α- producing cells in the CD4+ T cell population were significantly decreased due to HCA intervention (Figure 10b), while their incidences in CD8+ T cells were not affected in most of the tested cases (Figure 10c).

### 3.5. HCA Relieves DSS-Induced Colitis in Mice

**Figure 11:**
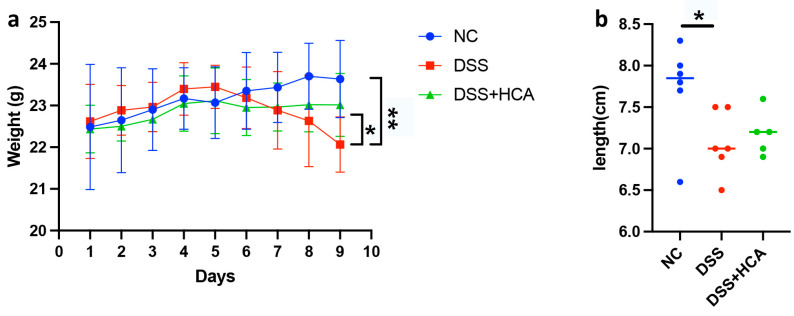
HCA attenuates DSS-induced colitis. (**a**) Body weight changes during the course of the experiment. (**b**) The colon lengths of mice as measured at sacrifice on day nine. Comparisons among the control, DSS, and DSS + HCA groups were performed using Mann–Whitney tests. * *p* < 0.05; ** *p* < 0.01. NC: normal control; DSS: dextran sodium sulfate; HCA: hyocholic acid.

We used the DSS-induced mouse colitis model to further access the potential of HCA against colonic inflammation. We induced colitis in C57BL/6 mice by administering 3% DSS in drinking water for nine days. HCA was administrated on day four via intraperitoneal injection for five days at a daily dose of 20 mg/kg body weight. DSS-induced body weight loss was relieved by HCA administration (Figure 11a). On day nine, the average colon length was longer in the HCA-treated mice than in the DSS group, but a statistical difference was not achieved (Figure 11b). Thus, HCA treatment could alleviate DSS-induced colonic colitis in mice to a certain extent, suggesting a potential protective/anti-inflammatory effect of HCA.

## 4. Discussion

EEN was first introduced to treat active IBD in the 1970s and became the frontline therapy in the 1990s [33]. Current evidence indicates that EEN improves outcomes in CD patients by ameliorating micronutrient and macronutrient deficiencies, reducing antigenic load, exerting direct anti-inflammatory effects, and correcting gut microbiota dysbiosis [16,34]. However, the mechanisms of action of EEN remain elusive. In this study, we reported the therapeutic outcomes of EEN in pediatric CD, characterized the differences in gut microbiota and the altered BA pool in pediatric CD patients during EEN therapy, and explored the effect of EEN-enriched HCA in inhibiting the inflammatory response. After eight weeks of EEN therapy, 91.7% (11/12) of pediatric CD patients achieved clinical remission, and 75% (9/12) achieved endoscopic remission, which was similar to previous studies [10,13,15]. EEN treatment significantly decreased serum and fecal inflammatory markers, including ESR, WBC, PLT, CRP, and FCP. Although BMI and STAMP scores displayed no significant improvement, other nutritional indexes (i.e., HB, ALB, and PA) significantly increased after EEN. Overall, our data revealed that EEN could induce clinical remission and endoscopic remission, decrease inflammatory markers, and partially improve the nutrition status of pediatric CD patients.

IBD-associated gut microbiota dysbiosis and impaired microbiota enzymatics lead to dysmetabolism, including reduced SCFA production and modifications in luminal BA pool composition [5,35]. Gut microbiota dysbiosis was repeatedly reported in pediatric CD patients and recognized as a crucial factor involved in the inflammation process and affecting the responses to multiple therapeutics [36]. In line with previous findings, the gut microbiota of pediatric CD patients showed decreased biodiversity, reduced levels of beneficial taxa such *Bifidobacterium* spp. and *Faecalibacterium prausnitzii*, and increased levels of proinflammatory bacteria such as *Escherichia.* Moreover, a reduced abundance of multiple BA-metabolizing bacteria, such as *Eubacterium* (which possess 7α-dehydroxylation) and *Ruminococcus* (which exhibit both 7α- and 7β- hydroxysteroid dehydrogenase (HSDH)) was detected in accordance with declined fecal levels of secondary and unconjugated BAs, confirming impaired BA metabolism. Our previous study suggested that the effects of anti-TNF agents for CD might be partially mediated by upregulating gut bacteria that produce bile salt hydrolases (BSH), thereby restoring BA metabolism and inhibiting inflammation [35]. In the present study, we observed that a range of BA-metabolizing bacteria increased in the gut microbiota of pediatric CD patients after EEN treatment, including several species of *Clostridium* (*C*. *innocuum*, *C. difficile*, and *C. perfringens*). Notably, *Clostridium* spp. are carriers of a range of genes involved in BA deconjugation and dehydroxylation [37]. For example, *C. innocuum* carries genes encoding enzymes 3β- HSDH, 7α-HSDH, 7β-HSDH and 5β-reductase (5BR) [38,39], thereby participating in BA transformations such as the production of oxo (keto) BAs. *Gordonibacter pamelaeae* and *Hungatella hathewayi*, two additional bacterial species increased under EEN, were also suggested to carry 3α/3β-HSDH and 5BR [39], respectively. Thus, EEN increased certain bacteria with enzymes involved in the production of these enriched BAs.

Gut microbiota dysbiosis in IBD can result in a profound impact in BA metabolism, including deficiencies in the deconjugation, dehydrogenation, and dehydroxylation of primary BAs. Altered BA metabolism, especially a reduced level of secondary BAs, is suggested to contribute to a pro-inflammatory state [21]. Deficiencies in DCA, LCA, and their metabolites, such as oxo derivatives, were suggested to promote intestinal inflammation in UC patients [40]. Duboc et al. reported increased levels of fecal conjugated BAs in active IBD, while secondary BAs were decreased [5]. Previous studies showed that some secondary bile acids, such as isoalloLCA, LCA, UDCA, and tauroursodeoxycholic acid (TUDCA), play important roles in maintaining intestinal homeostasis [41,42,43]. Connors et al. noted that patients who achieved and sustained remission after EEN had a fecal BA profile dominated by secondary BAs [44]. Our analysis also indicated a significant increase in secondary BA and unconjugated BA concentrations after EEN therapy. In addition, there were multiple EEN-enriched BAs that showed close associations with improvements in CD patients’ inflammatory and nutritional indexes. Interestingly, we found that the concentration of HCA increased and persisted after eight weeks of EEN in pediatric CD patients, and HDCA also significantly increased after eight weeks EEN.

Previous studies suggested that some BAs could regulate host immune responses by modulating the Th17 and Treg balance [22,45]. On this basis, we attempted to explore the role of HCA in suppressing intestinal inflammatory immune responses. HCA was recently found to simultaneously activate TGR5 while inhibiting FXR in enteroendocrine cells [46], and both receptors were found to modulate the inflammation occurring in IBD [47]. CD has been associated with an inflammatory CD4+ T cell phenotype [48]. Our data showed an anti-inflammatory effect of HCA through inhibiting TNF-α production in human PBMCs stimulated with anti-CD/anti-CD28 monoclonal antibodies, which revealed the role of HCA in attenuating the inflammatory response. Moreover, the administration of HCA alleviated DSS-induced acute colitis in mice, suggesting a potential protective/anti-inflammatory effect of HCA. Further experiments are required to access the anti-inflammatory capacities of other EEN-enriched BAs, and the results may lead to targets for the development of new therapies for IBD.

Recent studies indicate that BA receptors are involved in regulating both innate and adaptive immunity [49,50,51]. We found that some EEN-enriched BAs in CD patients are agonists/antagonists of some BA receptors, such as FXR, TGR5, VDR, and RORγt (Supplementary Table S2). TGR5 activation promotes the differentiation of macrophages towards M2 phenotypes, thereby enhancing the development of Treg cells via IL-10 production and inhibiting the M1 phenotype, which is known to produce pro-inflammatory cytokines [52]. Activating FXR via its agonist was also shown to prevent gut barrier dysfunction [53]. FXR knockout mice developed an IBD-like phenotype and exhibited pronounced hepatic inflammation and fibrosis [54]. Among the BAs enriched after EEN treatment, CA and CDCA are agonists of FXR while the MACs including αMCA, βMCA, and HCA were found to inhibit FXR as antagonists [46,49]. In our previous study, *B. longum* CECT 7894 improved the efficacy of infliximab for DSS-induced colitis and increased the expression of FXR via regulating the gut microbiota composition and bile acid metabolism [55]. Vitamin D deficiency is associated with the onset and activity of IBD, mainly because vitamin D exerts a regulatory role in mucosal immunity and host defenses via VDR [56]. As a subset of CD4+ T cells, Th17 cells produce IL-17A, IL-17F, IL-21, IL-22, IL-26, and the chemokine CCL20, which are critically involved in the mucosal inflammation observed CD. The oxo- (or keto-) derivatives of BAs are shown to be antagonists of RORγt [21], and the EEN-enriched 6-keto-LCA and 6,7-diketo-LCA may inhibit the proinflammatory activities of Th17 cells in a RORγt-dependent manner. Taken together, these EEN-enriched BAs and the BA receptors they activate/inhibit are versatile in modulating the development and functions of the innate and adaptive immune system and thereby contributing to colitis remission and mucosa repair in pediatric CD patients.

This study has some limitations. First, this study was conducted at a single center with a relatively small cohort. Multicenter randomized controlled trials could be performed to minimize confounding factors. Second, the assessment of gut microbiota and BAs was not performed in sustained and non-sustained remission CD patients after EEN therapy. Third, for research on the mechanism by which EEN enriches BAs, we only demonstrated that HCA could inhibit pro-inflammatory cytokine production by regulating CD4^+^ T cells in vitro. Thus, further experiments are required to explore and detect which BA receptors HCA stimulates/inhibits to perform its immune regulatory functions.

## 5. Conclusions

In summary, we showed that EEN therapy altered microbiota composition and the BA pool in pediatric CD patients. EEN treatment induced remission in children with active CD partially by increasing a number of secondary BAs, such as HCA, which could suppress TNF-α production by CD4+ T cells in PBMCs of CD patients and attenuate DSS-induced acute colitis in mice. These BAs are likely produced by increased levels of certain bacteria, including *Clostridium innocuum* and *Hungatella hathewayi,* which express 3β-hydroxysteroid dehydrogenase and 5β-reductase. Thus, certain BAs may provide a possibility for improving anti-inflammatory and immunity modulation effects in pediatric CD patients in the future.

## Figures and Tables

**Table 1:** Demographic and clinical characteristics of the pediatric CD patients before and after EEN treatment.

Characteristic	BSL	8 W	*p* Value ^1^	*q* Value ^2^
**Age, year, median (IQR)**	13 (11, 13)	/		
**Male, n (%)**	8 (66.7)	/		
**BMI, median (IQR)**	13.7 (12.8, 15.4)	15.4 (13.8, 18.0)	0.1135	0.1202
**STAMP, median (IQR)**	4.5 (2, 6.5)	3 (3, 6)	>0.99	0.99
**Disease location (ParisL)**				
L3, n (%)	10 (83.3)			
L4a, n (%)	2 (16.7)			
**PCDAI, median (IQR)**	38.8 (21.9, 44.4)	4.5 (2, 5)	<0.0001	0.0004
**CDEIS, median (IQR)**	14 (13, 15)	7 (3.8, 12)	0.0008	0.0021
**Laboratory values**				
ESR (mm/h), median (IQR)	109.5 (93.3, 120)	23 (9, 32)	<0.0001	0.0004
WBC (×10^9^/L), median (IQR)	11.9 (7.6, 13.4)	6.3 (5.0, 7.9)	0.0014	0.0025
HB (g/L), median (IQR)	98 (86.8, 105)	117.5 (111.3, 126)	<0.0001	0.0004
PCV (%), median (IQR)	32.6 (30.6, 34.5)	36.85 (34.3, 38.2)	0.0013	0.0025
PLT (×109/L), median (IQR)	522.5 (479.8, 552.8)	324 (261.3, 352.5)	0.0012	0.0025
CRP (mg/L), median (IQR)	42 (26, 73.5)	5 (5, 5)	<0.0001	0.0004
ALB (g/L), median (IQR)	31.5 (24.0, 34.7)	41.11 (40.5, 44.2)	<0.0001	0.0004
PA (mg/dL), median (IQR)	73 (53.1, 130.6)	216 (170.6, 256.6)	0.0015	0.0025
TRF (g/L), median (IQR)	1.7 (1.2, 2.3)	3.0 (2.8, 3.3)	0.0007	0.0021
TG (mmol/L), median (IQR)	1.0 (0.8, 1.2)	1.3 (1.1, 1.4)	0.033	0.045
TC (mmol/L), median (IQR)	2.9 (2.8, 3.9)	4.17 (3.6, 4.5)	0.035	0.045
HDL (mmol/L), median (IQR)	1.0 (0.8, 1.1)	1.1 (1.0, 1.2)	0.1081	0.1202
LDL (mmol/L), median (IQR)	1.7 (1.5, 1.7)	2.6 (2.0, 3.0)	0.0947	0.1136
FCP (μg/g), median (IQR)	1800 (1800, 1800)	410 (264.5, 1275)	0.032	0.045
**Outcome of EEN therapy**				
CR, n (%)		11(91.7)		
ER, n (%)		9 (75.0)		

CD, Crohn’s disease; EEN, exclusive enteral nutrition; HC, healthy controls; BSL, baseline; IQR, interquartile range; BMI, Body Mass Index; STAMP, Screening Tool for the Assessment of Malnutrition in Pediatrics; PCDAI, Pediatric Crohn’s Disease Activity Index; CDEIS, Crohn’s Disease Endoscopic Index of Severity; ESR, erythrocyte sedimentation rate; WBC, white blood cells; HB, hemoglobin; PCV, packed cell volume; PLT, platelets; CRP, c-reactive protein; ALB, albumin; PA, prealbumin; TRF, transferrin; TG, triglyceride; TC, total cholesterol; HDL, high-density lipoprotein; LDL, low-density lipoprotein; FCP, fecal calprotectin; CR, clinical remission (PCDAI < 12 at weeks 8); ER, endoscopic remission (CDEIS < 10 at weeks 8). ^1^ The data were compared via the nonparametric Mann–Whitney U test. ^2^ *p*-values were adjusted with the Benjamini–Hochberg correction procedure for controlling false discovery rate.

## Data Availability

The data that support the findings of this study are available from figshare at the DOI https://doi.org/10.6084/m9.figshare.19665972.v1. The raw data from this study will be published after acceptance of this paper for publication.

## Supplementary Materials

The following supporting information can be downloaded at: https://www.mdpi.com/article/10.3390/nu14214463/s1, Supplementary Table S1. Clinical characteristics of PBMC donors with active CD. Supplementary Table S2. EEN-enriched bile acids are natural ligands of various receptors that are distributed across different immune cells. Supplementary Figure S1. EEN-induced endoscopic improvement in pediatric CD. Endoscopic images showing the transverse colon in two patients and the ileocecal junction in one patient at baseline and eight weeks after EEN treatment. Supplementary Figure S2. Metagenomic analysis of the gut microbiota profiles in CD patients before and after EEN induction therapy. Heatmap demonstrates changes in the relative abundances of gut bacteria at the genus level during eight weeks of EEN induction therapy. Significance was determined by Mann–Whitney U test with *p* < 0.05 based on the clr-transformed counts. # Significantly different between CD and HC at baseline. * Significantly changed after either 4 w or 8 w of EEN therapy. Supplementary Figure S3. EEN-induced changes in gut microbiota and bile acid metabolism are closely associated with CD symptom improvement. Heatmap showing the Spearman correlations between the serum inflammatory markers and severity indexes of pediatric CD (PCDAI and CDEIS), and (a) BAs and (b) gut bacterial species. Significance was determined by *t*-test. * *p* < 0.05; ** *p* < 0.01; *** *p* < 0.001.
