# Supplementary material for: Exclusive Enteral Nutrition Exerts Anti-Inflammatory Effects through Modulating Microbiota, Bile Acid Metabolism, and Immune Activities
Source: Nutrients. 2022 Oct 24;14(21):4463. doi: 10.3390/nu14214463 (PMC9657881; doi:10.3390/nu14214463)
Supplement: Supplementary file 1 [file nutrients-14-04463-s001.zip › Supp_Table_S1.pdf]

Supplementary Table S1. Clinical characteristics of PBMC donors with active CD.

	**Sex**	**Age**	**Time of onset**	**Maintenance treatment**
P1	Male	16	1 year	Infliximab
P2	Male	23	6 months	Mesalazine
P3	Male	23	1 years	Infliximab
P4	Male	26	10 years	Infliximab, Prednisolone
P5	Male	30	4 years	Infliximab, Mesalazine

CD, Crohn's disease; PBMC, peripheral blood mononuclear cell.
