# Supplementary material for: Exclusive Enteral Nutrition Exerts Anti-Inflammatory Effects through Modulating Microbiota, Bile Acid Metabolism, and Immune Activities
Source: Nutrients. 2022 Oct 24;14(21):4463. doi: 10.3390/nu14214463 (PMC9657881; doi:10.3390/nu14214463)
Supplement: Supplementary file 1 [file nutrients-14-04463-s001.zip › Supp_Table_S2.pdf]

Supplementary Table S2. EEN−enriched bile acids are natural ligands of various receptors which are distributed on different immune cells.

**Receptors**	**Agonists**	**Antagonists**	**Immune cell distribution**
G−protein bile acid receptor 1 (GPBAR1/Tgr5)	CA, CDCA, HCA, HDCA, UDCA, bUDCA, 6−ketoLCA		Macrophage
Farnesoid−X−Receptor (NR1H4)	CA, CDCA	αMCA, βMCA, HCA	Macrophage, Dendritic cell
Vitamin D receptor (NR1H1)	6−keoto−LCA, 6,7−diketoLCA		Macrophage, Dendritic cell
Retinoic acid−related orphan receptor−γt (NR1F1−3)	6−keoto−LCA, 6,7−diketoLCA		T helper 17, group 3 innate lymphoid cells

EEN, exclusive enteral nutrition; CA, cholic acid; CDCA, chenodeoxycholic acid; HCA, hyocholic acid; HDCA, α−hyodeoxycholic acid; UDCA, ursodeoxycholic acid; bUDCA, 3β−ursodeoxycholic acid; 6−ketoLCA, 6−ketolithocholic acid; aMCA, α−muricholic acid; βMCA, β−muricholic acid; HCA, hyocholic acid; 6,7−diketoLCA, 6,7−diketolithocholic acid.
