# Supplementary figures and images for: Exclusive Enteral Nutrition Exerts Anti-Inflammatory Effects through Modulating Microbiota, Bile Acid Metabolism, and Immune Activities
Source: Nutrients. 2022 Oct 24;14(21):4463. doi: 10.3390/nu14214463 (PMC9657881; doi:10.3390/nu14214463)

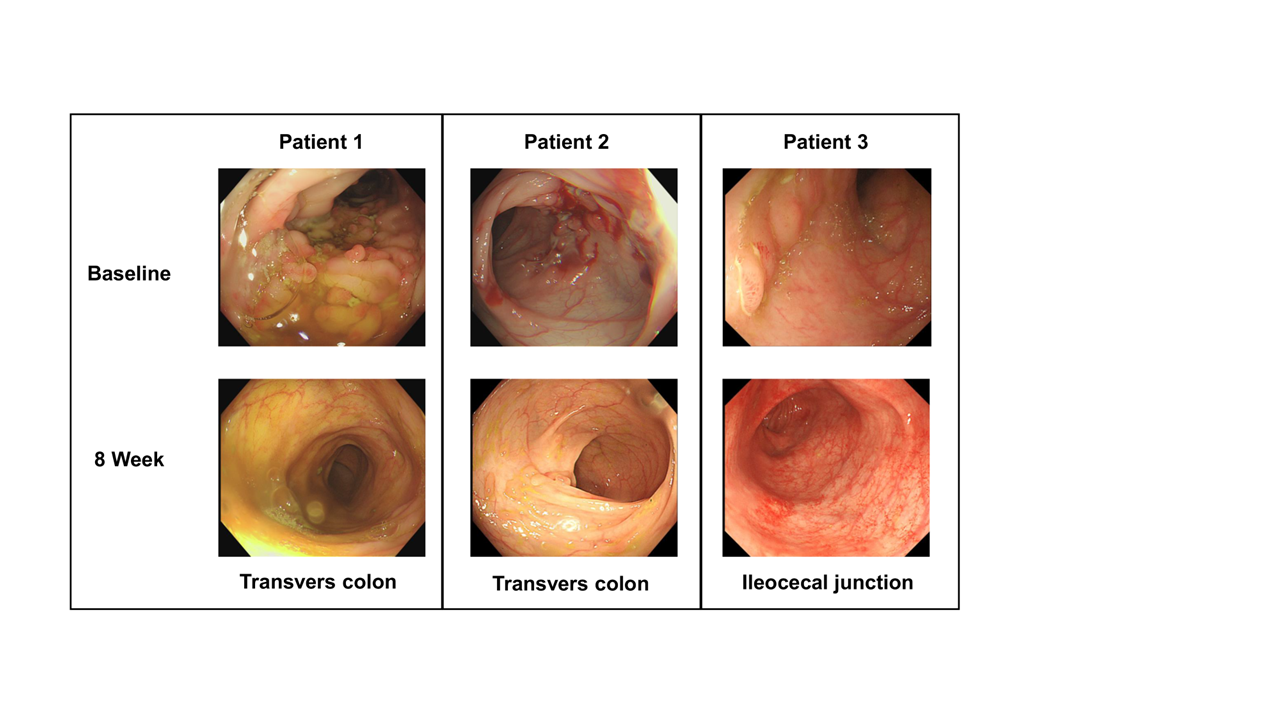

Supplement: Supplementary file 1 [file nutrients-14-04463-s001.zip › Figure S1.tif]

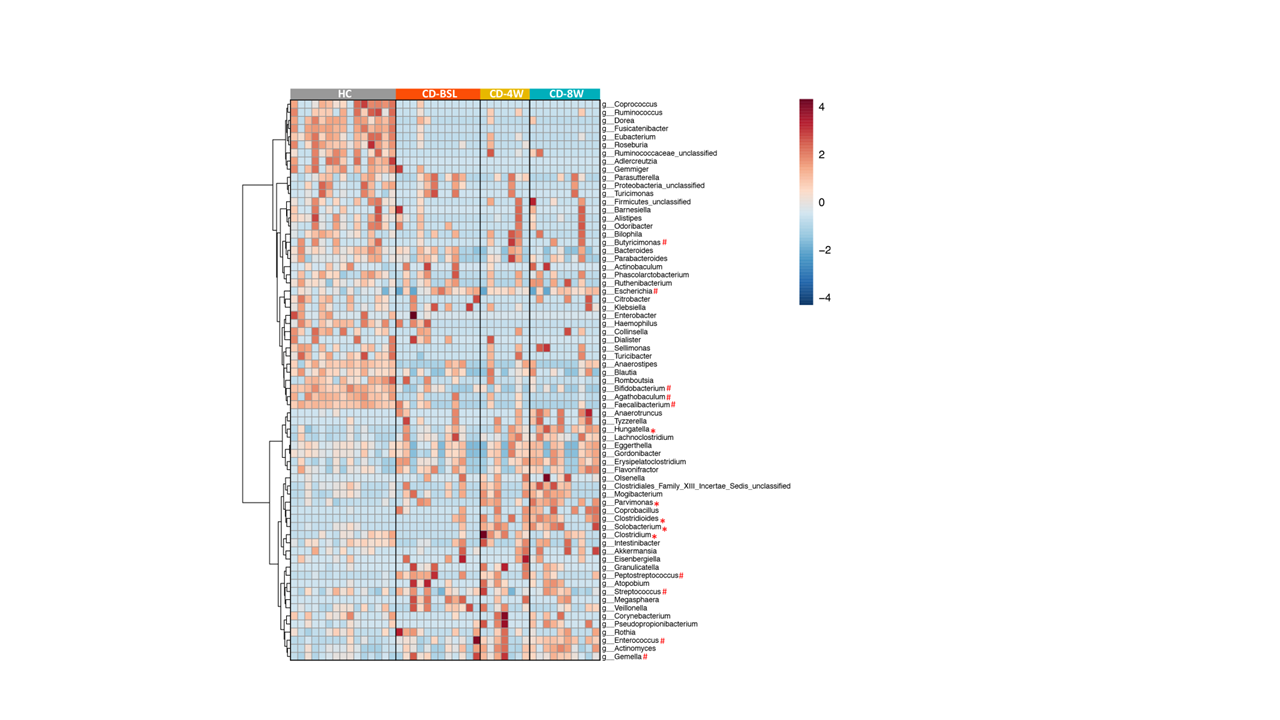

Supplement: Supplementary file 1 [file nutrients-14-04463-s001.zip › Figure S2.tif]

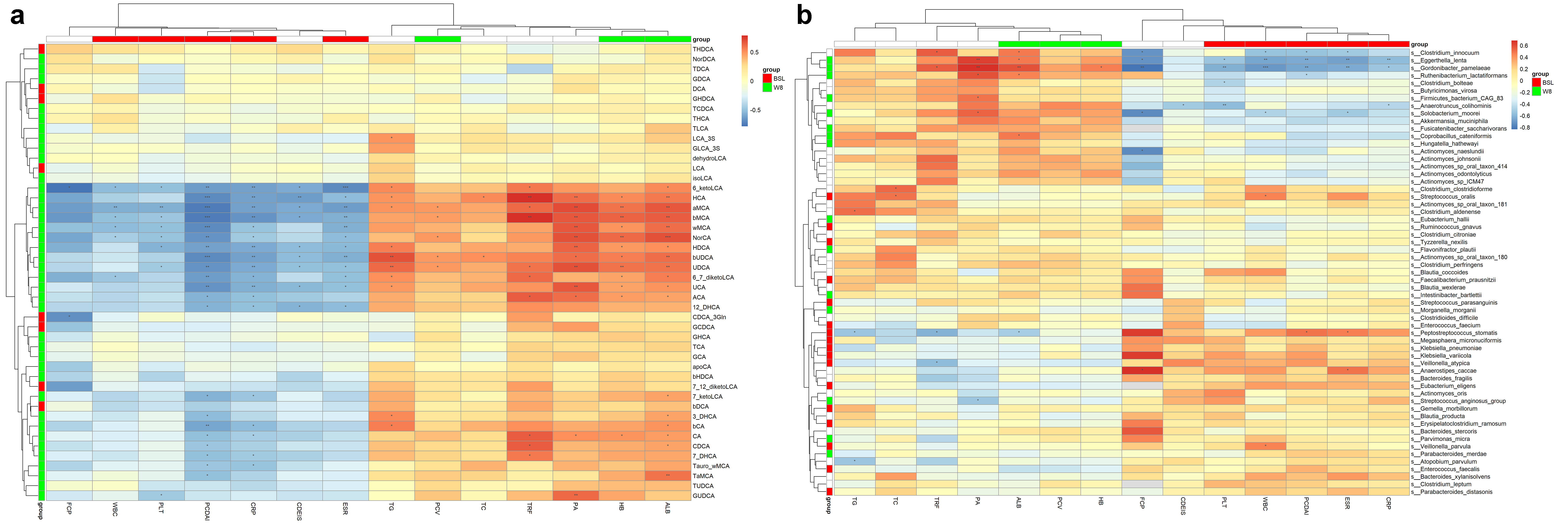

Supplement: Supplementary file 1 [file nutrients-14-04463-s001.zip › Figure S3.tif]
